# Adjustment of positive end‐expiratory pressure based on body mass index during general anaesthesia: a randomised controlled trial*

**DOI:** 10.1111/anae.16656

**Published:** 2025-06-23

**Authors:** Helene Selpien, Jann Penon, David Thunecke, Dirk Schädler, Ingmar Lautenschläger, Henning Ohnesorge, Christine Eimer, Caroline Wolf, Armin Sablewski, Tobias Becher

**Affiliations:** ^1^ Department for Anaesthesiology and Intensive Care Medicine University Hospital Schleswig‐Holstein, Campus Kiel Germany

**Keywords:** body mass index, lung ultrasound, lung‐protective ventilation, positive end‐expiratory pressure, postoperative pulmonary complications

## Abstract

**Introduction:**

Lung‐protective ventilation is essential for preventing postoperative pulmonary complications. While maintaining a low driving pressure and optimising PEEP is of importance, the ideal strategy remains contentious. This study evaluated whether adjusting PEEP based on BMI, compared with standard PEEP, could reduce driving pressure and peri‐operative loss of lung aeration.

**Methods:**

We conducted a randomised controlled, patient‐blinded, single‐centre superiority trial with two parallel groups. Adult patients undergoing surgery with general anaesthesia who required tracheal intubation were assigned randomly to either standardised PEEP (PEEP = 5 cmH_2_O; group PEEP‐5) or PEEP set according to BMI (PEEP = BMI/3 cmH_2_O; group PEEP‐BMI/3). Patients' lungs were ventilated using a volume‐controlled mode with tidal volumes of 7 ml.kg^‐1^ predicted body weight. Lung aeration scores were assessed using ultrasound pre‐ and postoperatively.

**Results:**

Sixty patients were enrolled and allocated randomly. Adjustment of PEEP according to BMI/3 was associated with a significantly lower driving pressure, with a median (IQR [range]) of 8.9 (7.1–10.4 [5.2–14.9]) cmH_2_O in group PEEP‐5 and 7.9 (7.2–8.5 [5.9–14.1]) cmH_2_O in group PEEP‐BMI/3 (p = 0.027) and higher mean (SD) respiratory system compliance (group PEEP‐5, 0.83 (0.20) ml cmH_2_O^‐1^ kg^‐1^ predicted body weight vs. group PEEP‐BMI/3, 0.95 (0.17) ml cmH_2_O^‐1^ kg^‐1^ predicted body weight; p = 0.020). Lung ultrasound revealed a reduced postoperative loss of lung aeration in patients allocated to the BMI/3 group. Patients allocated to the BMI‐adjusted group required less supplemental oxygen, had less newly developed atelectasis and had higher oxygen saturations upon arrival in the post‐anaesthesia care unit.

**Discussion:**

In patients without major pulmonary disease who were undergoing non‐cardiothoracic surgeries with tracheal intubation, adjusting PEEP based on a calculation of BMI/3 improved lung mechanics and reduced postoperative loss of lung aeration. This approach provides a straightforward and pragmatic method for individualising PEEP in patients undergoing general anaesthesia.

## Plain Language Summary

Protecting the lungs during surgery is important to stop problems with breathing afterwards. One way to do this is by using the right air pressure when helping someone breathe with a machine. But doctors don't all agree on the best way to do this. This study looked at whether using air pressure based on a person's body size (BMI) is better than using the same air pressure for everyone. We did a study with two groups of adult patients who were having surgery and needed help breathing with a tube and machine. Some patients got the same air pressure (5 cmH_2_O), while others got air pressure based on their BMI (BMI divided by 3, about 7‐10 cmH_2_O). We used a special setting on the breathing machine that gave each person the same amount of air based on their body size. We used ultrasound to check how well their lungs were working before and after surgery. We studied 60 people. The group that got air pressure based on BMI had better results. Their lungs needed less pressure to work properly, and they had better lung movement. Their lungs also stayed more open after surgery. They needed less extra oxygen, had fewer new breathing problems, and had better oxygen levels after surgery. In people without serious lung problems, using air pressure based on BMI during surgery helped their lungs work better and kept them healthier afterward. This method is simple and can be a good way for doctors to choose the best air pressure for each patient during surgery.

## Introduction

Postoperative pulmonary complications following mechanical ventilation are associated with poorer patient outcomes, including increased morbidity, mortality and prolonged hospital stays, thereby contributing to a higher economic burden [1, 2]. The incidence rates of postoperative pulmonary complications vary depending on the definition, ranging from 2.0% to 5.6% in the general surgical population to as high as 40% in patients having thoracic surgery [3, 4]. Given this high incidence, reducing the occurrence of postoperative pulmonary complications is of critical importance and warrants targeted interventions to improve peri‐operative outcomes [5].

Previous studies have shown that a lung‐protective ventilation strategy combining low tidal volumes [6, 7], moderate positive end‐expiratory pressure (PEEP) [6, 8] and intermittent recruitment manoeuvres [6, 9] reduces the incidence of postoperative pulmonary and extrapulmonary complications significantly compared with a non‐protective ventilation strategy with high tidal volume and no PEEP [6]. However, the relative contribution of individual components of lung‐protective ventilation to the improved outcomes remains unclear. Controversy persists regarding the optimal PEEP settings during general anaesthesia. Studies in which different PEEP levels were assigned randomly without tailoring them to individual patient characteristics showed no significant difference in outcomes between higher and lower PEEP settings [10, 11]. Recent evidence suggests that adjusting ventilatory settings to reduce the pressure difference (driving pressure) between end‐inspiratory plateau pressure and PEEP may mitigate the risk of postoperative pulmonary complications, with growing evidence suggesting that individualised PEEP may be more effective than fixed levels [12, 13, 14, 15, 16]. Various approaches to individualising PEEP based on, for example, transpulmonary pressure, decremental PEEP trials and electrical impedance tomography have been explored, but still present challenges regarding availability, time and feasibility in routine anaesthetic practice [17, 18, 19, 20, 21].

We found that in healthy individuals undergoing general anaesthesia in a supine position, the PEEP required to prevent both pulmonary overdistension and alveolar collapse, as assessed with electrical impedance tomography, correlated positively with the patient's BMI and corresponded to approximately one‐third of the BMI [22]. Consequently, we hypothesised that adjusting PEEP to approximately one‐third of a patient's BMI could provide a pragmatic and easy‐to‐implement approach to reducing driving pressure, thereby alleviating alveolar overdistension and atelectasis without necessitating additional devices, monitoring or time‐consuming titration manoeuvres.

## Methods

This randomised controlled, patient‐blinded, single‐centre superiority trial with two parallel groups took place at the central operating theatres of University Medical Centre Schleswig‐Holstein, Campus Kiel, Germany. The study protocol received approval from the ethics committee and has been published previously [23]. All patients provided written informed consent and received treatment in accordance with the Declaration of Helsinki.

Patients were screened prospectively at the pre‐operative anaesthesia clinic for study inclusion. Adult patients scheduled for elective surgery under general anaesthesia requiring tracheal intubation, with an expected duration of anaesthesia > 2 h, were eligible to participate. Non‐cardiothoracic, non‐neurological procedures performed by the general, vascular, orthopaedic, gynaecological, urological and plastic surgery departments were included. We did not study the following patients: those having planned facemask or supraglottic airway device ventilation; laparoscopic or thoracic surgery; surgery involving cardiopulmonary bypass; patients in positions exceeding 10° Trendelenburg/reverse‐Trendelenburg position; patients who were pregnant; BMI > 60 kg.m^‐2^; those with acute cardiac decompensation and severe pulmonary conditions (e.g. acute respiratory distress syndrome, pulmonary fibrosis, pneumonia, chronic obstructive pulmonary disease); and patients unable to give informed consent.

We collected baseline characteristics including age; height; weight; sex; and waist‐to‐hip ratio. Before induction of general anaesthesia, patients underwent a complete lung ultrasound examination covering 12 thoracic zones, as described by Monastesse et al. [24]. Ultrasound videos were stored electronically for post‐hoc analysis. After induction of anaesthesia, group allocation was determined by opening a sealed numbered opaque envelope. Randomisation was performed using random permuted blocks with a 1:1 allocation ratio and 11 blocks of variable sizes. The sequence within each block and the order of the blocks themselves were randomised by a designated team member (using www.randomizer.org), who was not involved in patient recruitment or data collection. All other team members and patients remained blinded to the randomisation sequence.

Initially, all patients were ventilated in a volume‐controlled mode with tidal volume of 7 ml.kg^‐1^ predicted body weight, PEEP of 5 cmH_2_O and end‐inspiratory pause of 0.25–0.50 s to ensure continuous reliable assessment of driving pressure. After arrival in the operating theatre, PEEP was either maintained at 5 cmH_2_O in the control group (group PEEP‐5) or adjusted to BMI/3 cmH_2_O in the intervention group (group PEEP‐BMI/3). One sustained‐inflation recruitment manoeuvre was performed following PEEP adjustment, applying an airway pressure of 20 cmH_2_O above PEEP for the intervention group and a fixed airway pressure of 30 cmH_2_O for the control group, both for a duration of 10 s. According to protocol, the same recruitment manoeuvres were performed following a ventilator circuit disconnection and in instances where oxygen saturation declined < 90% despite ventilation with a fraction of inspired oxygen > 60%. Tidal volume was maintained constant at 7 ml.kg^‐1^ predicted body weight for the entire duration of mechanical ventilation. According to our departmental standard, neuromuscular blockade was assessed using train‐of‐four monitoring in all patients, and a T4:T1 ratio of > 0.9 was required before tracheal extubation.

Following tracheal extubation, patients were transferred to either the post‐anaesthesia care unit (PACU) or the ICU, based on the pre‐operative risk assessment for extended postoperative care [25]. On arrival in the PACU or ICU, SpO_2_ was determined without additional oxygen supplementation. Oxygen supplementation was initiated to maintain SpO_2_ > 90%. A second lung ultrasound examination was performed in the PACU or ICU. In patients with a tracheal tube in situ upon transfer to the ICU, PEEP was maintained as set in the operating theatre and the second lung ultrasound was performed after tracheal extubation.

The study intervention was terminated in instances of uncontrollable haemodynamic/respiratory instability or when acute cardiac or pulmonary decompensation occurred. Protocol adherence was monitored by a team member to ensure correct intra‐operative intervention by the treating clinician.

The primary endpoint was the average driving pressure during volume‐controlled ventilation with a tidal volume of 7 ml.kg^‐1^ predicted body weight after PEEP adjustment. Driving pressure was calculated as end‐inspiratory plateau pressure measured at the end of the inspiratory pause minus PEEP [26]. We focused on driving pressure due to its clinical relevance to postoperative pulmonary complications [8, 12]. Secondary endpoints included: changes in lung aeration score between pre‐surgery and arrival in the PACU (assessed by lung ultrasound); intra‐operative respiratory system compliance (C_rs_); the elastic static (MP_el‐stat_) and elastic dynamic mechanical power (MP_el‐dyn_) of ventilation; intra‐operative fluid requirements and vasopressor requirements; number of hypotensive events (defined as mean arterial pressure < 65 mmHg for > 1 min); time‐weighted average of hypotensive events; number of intra‐operative alveolar recruitment manoeuvres (performed only if SpO_2_ < 90% with fraction of inspired oxygen >60%); first oxygen saturations measured after arrival in the PACU (without oxygen supplementation); number of patients requiring oxygen supplementation in the recovery room to maintain SpO_2_ ≥ 90%; number of patients with ≥1 postoperative pulmonary complication. Postoperative pulmonary complications were defined based on established criteria from previous trials as the postoperative onset of any of the following conditions: respiratory failure requiring non‐invasive or invasive mechanical ventilation; exacerbations of underlying chronic lung conditions; pleural effusion; atelectasis; bronchospasm; pulmonary oedema; pneumothorax; pneumonia; and acute respiratory distress syndrome [1, 3, 4, 27, 28, 29].

Ultrasound sequences were analysed independently by two members of the research team (HS, CE, CW, AS or TB), who were blinded to patient group allocation and each other's assessment. To further reduce the potential for subjectivity in the evaluation of the lung ultrasound data, the ultrasound videos were pseudonymised. Employing a lung aeration score modified for peri‐operative use as described by Monastesse et al. [24], newly developed atelectasis was defined as an increase in lung aeration score to 3 points in any examined lung area, indicating complete consolidation (online Supporting Information Table S1). Discordant ratings were reconciled by consensus with a third team member.

Using G*power 3.1 software [30, 31], we calculated a sample size of 54 (27 per group) patients, based on a significance level of p = 0.05 and a power (1‐β) of 0.8 with an assumed mean (SD) driving pressure of 9 (3) cmH_2_O in the control group and of 7 (2) cmH_2_O in the intervention group. The calculated sample size was increased by 10% to compensate for technical difficulties and attrition, resulting in a total of 60 patients.

Data were assessed for normal distribution using the Shapiro–Wilk test. For continuous variables, between‐group differences were examined using a two‐tailed unpaired t‐test for parametric data and the Mann–Whitney test for non‐parametric data. If heteroscedasticity was detected, Welch's t‐test was used to account for unequal variances between groups. Categorical variables were analysed using Fisher's exact test. For respiratory mechanics (driving pressure, C_rs_, mechanical power), the average intra‐operative value between PEEP adjustment and tracheal extubation was calculated for each patient and used for subsequent analyses. For the secondary endpoint, ‘time‐weighted average of hypotension events’, the area under the curve of 65 mmHg for each event was calculated by summing the difference between mean arterial pressure and the threshold, multiplied by duration of the event. The total area under the curve was divided by the observed time for each patient, resulting in a time‐weighted average of hypotension per patient. Changes in lung aeration scores were evaluated using a two‐way ANOVA for repeated measures with time‐point (pre‐operative/postoperative) serving as one independent variable and study group (intervention/control) as the other.

The relationship between driving pressure and BMI was assessed for both groups with linear regression. To compare the slopes of two linear regression lines, we used the built‐in analysis of GraphPad Prism, which is equivalent to an analysis of covariance (ANCOVA) [32]. This method assesses whether the slopes of the regression lines differ significantly by fitting both datasets simultaneously and evaluating the statistical interaction between the grouping factor (PEEP strategy) and the relationship between BMI (independent variable) and driving pressure (dependent variable). A significant interaction indicates that the slopes differ between groups. A two‐tailed test was used to determine whether slopes and intercepts of the lines differed significantly between groups. All statistical analyses were conducted using GraphPad Prism version 10.0.0 for Windows (GraphPad Software, Boston, MA, USA). P values <0.05 were considered statistically significant.

## Results

**Figure 1:**
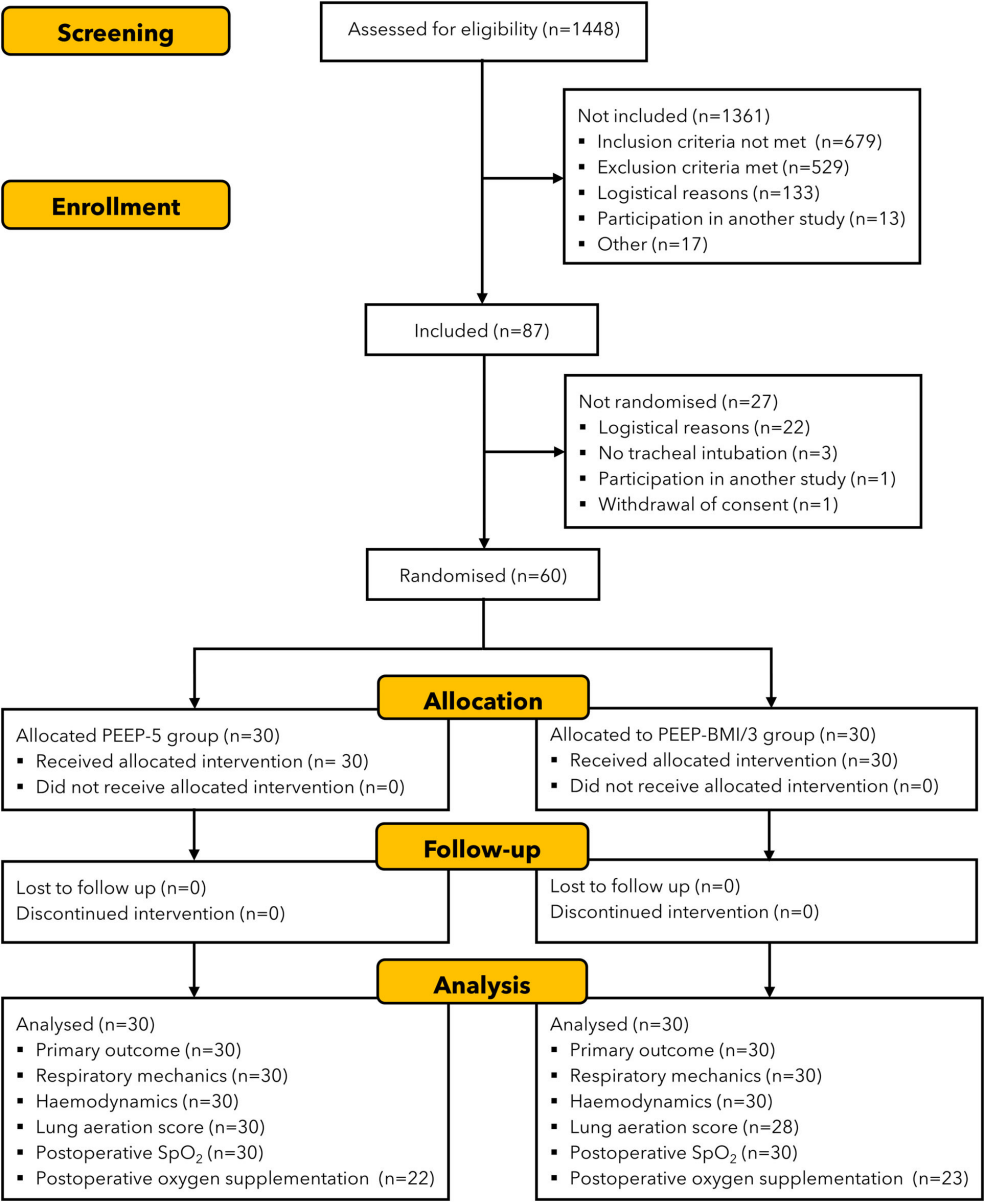
Study flow diagram.

Patient recruitment took place from 28 February 2023 to 26 July 2023. Sixty patients were allocated randomly and completed the intervention. Median (IQR [range]) age was 65 (55–74 [22–95]) y and mean (SD) BMI was 27.8 (4.8) kg.m^‐2^. Data for the primary endpoint, respiratory mechanics and haemodynamics were analysed for all included patients. For technical reasons, the postoperative lung aeration score could not be determined in two patients in the intervention group. Patient flow through the trial is shown in Fig. 1. Patient and clinical characteristics showed no statistically significant differences between groups (Table 1).

**Table 1:** Baseline patient characteristics. Values are median (IQR [range]) or mean (SD).

	PEEP‐5	PEEP‐BMI/3
	n = 30	n = 30
Age; y	64 (57–74 [32–95])	63 (54–74 [22–90])
Sex; male	19	16
Weight; kg	88 (17)	82 (20)
BMI; kg.m^‐2^	28.6 (4.4)	27.0 (5.2)
Duration of surgery; min	176 (127–223 [53–473])	175 (117–222 [51–421])
Duration of ventilation; min	247 (170–294 [134–555])	243 (184–287 [102–537])
PEEP; cmH_2_O	5 (5–5 [5–5])	9 (7–10 [7–13])
Balanced anaesthesia/TIVA; n	30/0	28/2
Cumulative sufentanil dose; μg	71 (29)	69 (41)
Type of surgery		
Visceral	21	14
Vascular	7	7
Orthopaedic	2	4
Gynaecological	0	3
Urological	0	1
Plastic	0	1
ASA physical status; n		
1	0	1
2	15	17
3	15	12
Exercise capacity ≥ 4 METs; n	26	23
ARISCAT score	26 (19–35 [3–65])	26 (18.75–34 [0–49])

TIVA, total intravenous anaesthesia; MET, metabolic equivalents; ARISCAT, Assess Respiratory Risk in Surgical Patients in Catalonia.

**Figure 2:**
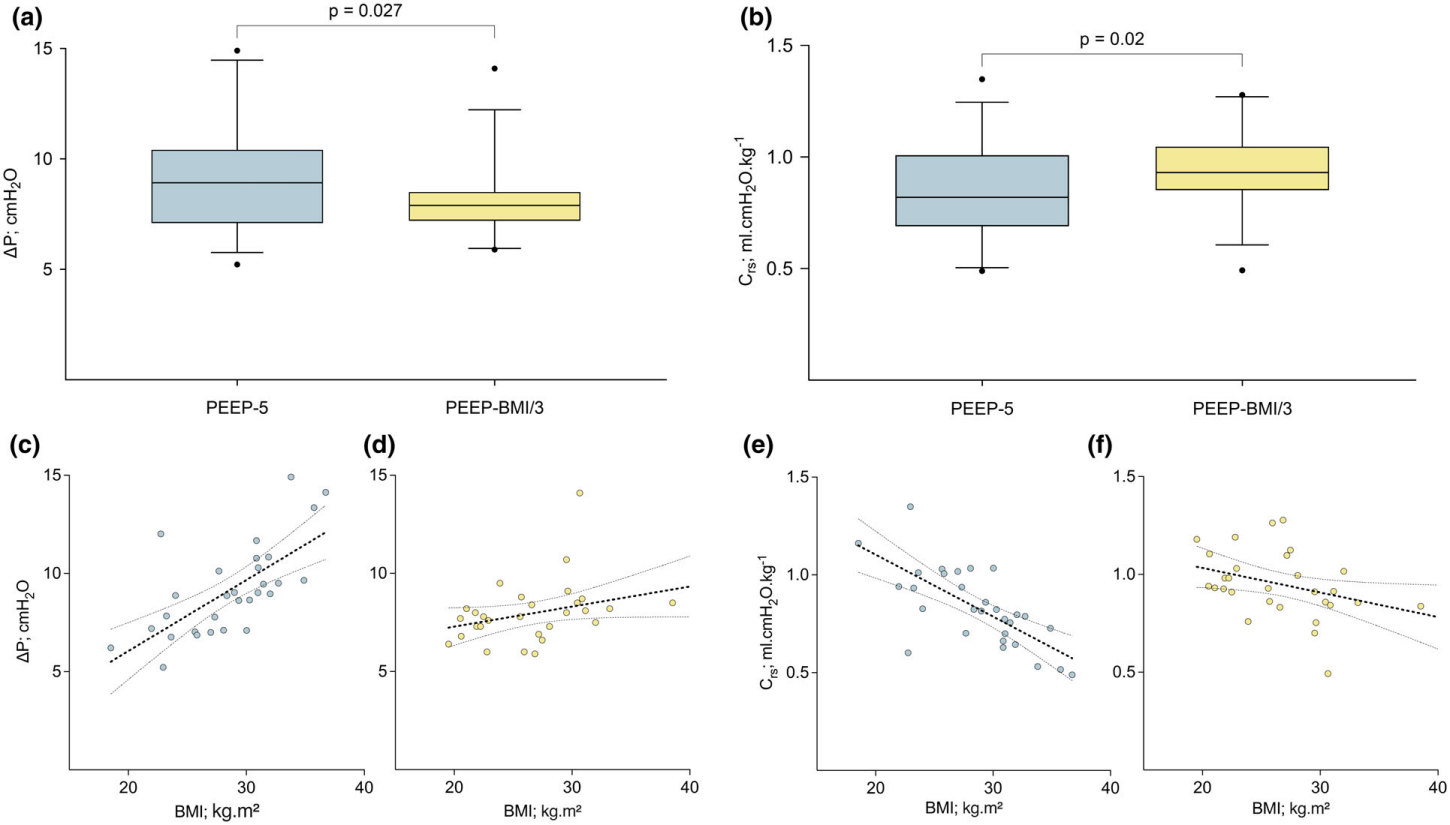
Results for driving pressure and C_rs_. Blue, group PEEP‐5; yellow, group PEEP‐BMI/3. Comparison of driving pressure (a) and C_rs_ (b) between PEEP‐5 cmH_2_O and PEEP‐BMI/3 cmH_2_O is illustrated as a box plot, which displays the median, 25th and 75th percentiles, whiskers indicating the 5th to 95th percentile, and dots representing outliers. Panels (c) and (d) display correlation between BMI and driving pressure for PEEP‐5 (c) and PEEP‐BMI/3 (d). Panels (e) and (f) display the correlation between BMI and C_rs_ for PEEP‐5 (e) and PEEP‐BMI/3 cmH_2_O (f). Dotted lines represent regression line. Upper and lower dotted lines mark the boundaries of the 95% confidence interval.

For the primary endpoint, a statistically significant reduction was found in driving pressure from a median (IQR [range]) of 8.9 (7.1–10.4 [5.2–14.9]) cmH_2_O in group PEEP‐5 to 7.9 (7.2–8.5 [5.9–14.1]) cmH_2_O in group PEEP‐BMI/3 (p = 0.027, Fig. 2a). Correspondingly, there was an increase in compliance from mean (SD) 0.83 (0.2) (95%CI 0.76–0.91) ml.cmH_2_O^‐1^.kg^‐1^ predicted body weight with PEEP‐5 to 0.95 (0.17) (95%CI 0.88–1.01) ml.cmH_2_O^‐1^.kg^‐1^ predicted body weight with PEEP‐BMI/3 (p = 0.020, Fig. 2b). The resulting driving pressure was correlated significantly with BMI for group PEEP‐5 but not for group PEEP‐BMI/3 (Fig. 2c and d), whereas the C_rs_ was correlated negatively with BMI for both groups (Fig. 2e and f). The positive correlation between driving pressure and BMI, as well as the negative correlation between C_rs_ and BMI, was significantly stronger with PEEP‐5 compared with PEEP‐BMI/3 (p = 0.006 for driving pressure and p = 0.027 for C_rs_, respectively), indicating that the negative influence of BMI on respiratory mechanics was attenuated with the PEEP‐BMI/3 strategy.

For further respiratory mechanics, MP_el‐stat_ was significantly lower in group PEEP‐5 (mean (SD) 0.052 (0.008) J.min^‐1^.kg^‐1^ predicted body weight) than in group PEEP‐BMI/3 (mean (SD) 0.098 (0.023) J.min^‐1^.kg^‐1^ predicted body weight, p = < 0.001). We found no significant between‐group differences for the other components of mechanical power (Table 2).

**Table 2:** Secondary endpoints. Continuous variables are presented as mean (SD) or median (IQR [range]). For categorical variables, numbers with absolute risk difference (ARD) and relative risk (RR) are reported.

	PEEP‐5	PEEP‐BMI/3	Difference of mean or median (95%CI)	p value
	n = 30	n = 30		
Primary outcome				
Driving pressure; cmH_2_O	8.9 (7.1–10.4 [5.2–14.9])	7.9 (7.2–8.5 [5.9–14.1])	‐1.0 (‐1.9 to ‐0.16)	0.027
Secondary outcomes (continuous)				
Compliance; ml.cmH_2_O^‐1^.kg^‐1^ predicted body weight	0.83 (0.2)	0.95 (0.17)	‐0.12 (‐0.21 to ‐0.02)	0.02
MP_el‐dyn_; j.min^‐1^.kg^‐1^ predicted body weight	0.045 (0.033–0.055 [0.024–0.101])	0.042 (0.037–0.048 [0.031–0.076])	‐0.002 (‐0.01–0.004)	0.523
MP_el‐stat_; j.min^‐1^.kg^‐1^ predicted body weight	0.052 (0.008)	0.098 (0.023)	0.05 (0.04–0.06)	< 0.0001
Fluid requirements; ml				
Crystalloids	1350 (500–2275 [300–4000])	1400 (875–2475 [200–8500])	‐260 (‐800–300)	0.33
Colloids	150 (100–275 [100–300])	200 (100–200 [100–1100])	0 (‐100–100)	0.78
Norepinephrine requirement; μg.kg^‐1^.min^‐1^	0.0437 (0–0.068 [0–0.124])	0.043 (0–0.089 [0–0.158])	0.003 (‐0.012–0.032)	0.478
Recruitment manoeuvres > 1	1 (1–1 [1–2])	1 (1–2 [1–4])	0 (0–0)	0.179
Number of hypotensive events	1 (0–3 [0–12])	3 (0–8 [0–20])	1 (0–3)	0.081
Time‐weighted average of hypotensive events; mmHg	0.1 (0–0.59 [0–1.75])	0.5 (0–1.3 [0–4.6])	0.5 (0–0.9)	0.057
Duration of PACU stay; min	240 (191–290 [150–895])	210 (130–270 [80–420])	‐30 (‐85–30)	0.281
SpO_2_ upon arrival in PACU; %	92 (4)	94 (3)	2 (0–4)	0.049
Secondary outcomes (categorical)				
Postoperative oxygen requirement for SpO_2_ ≥ 90%	13*	6^†^	ARD: 0.33 (0.04–0.62) RR: 2.27 (1.1–5)	0.036
New atelectasis; n	7	0	ARD: 0.23 (0.043–0.43) RR: ∞ (1.98 to ∞)	0.011
≥ 1 PPC	11	7	ARD: 0.13 (‐0.12–0.37) RR: 1.6 (0.73–3.5)	0.399

MP_el‐dyn_, elastic‐dynamic component of mechanical power; MP_el‐stat_, elastic‐static component of mechanical power; PPC, postoperative pulmonary complication; PACU, post‐anaesthesia care unit.

* n = 22.

^†^ n = 23.

**Figure 3:**
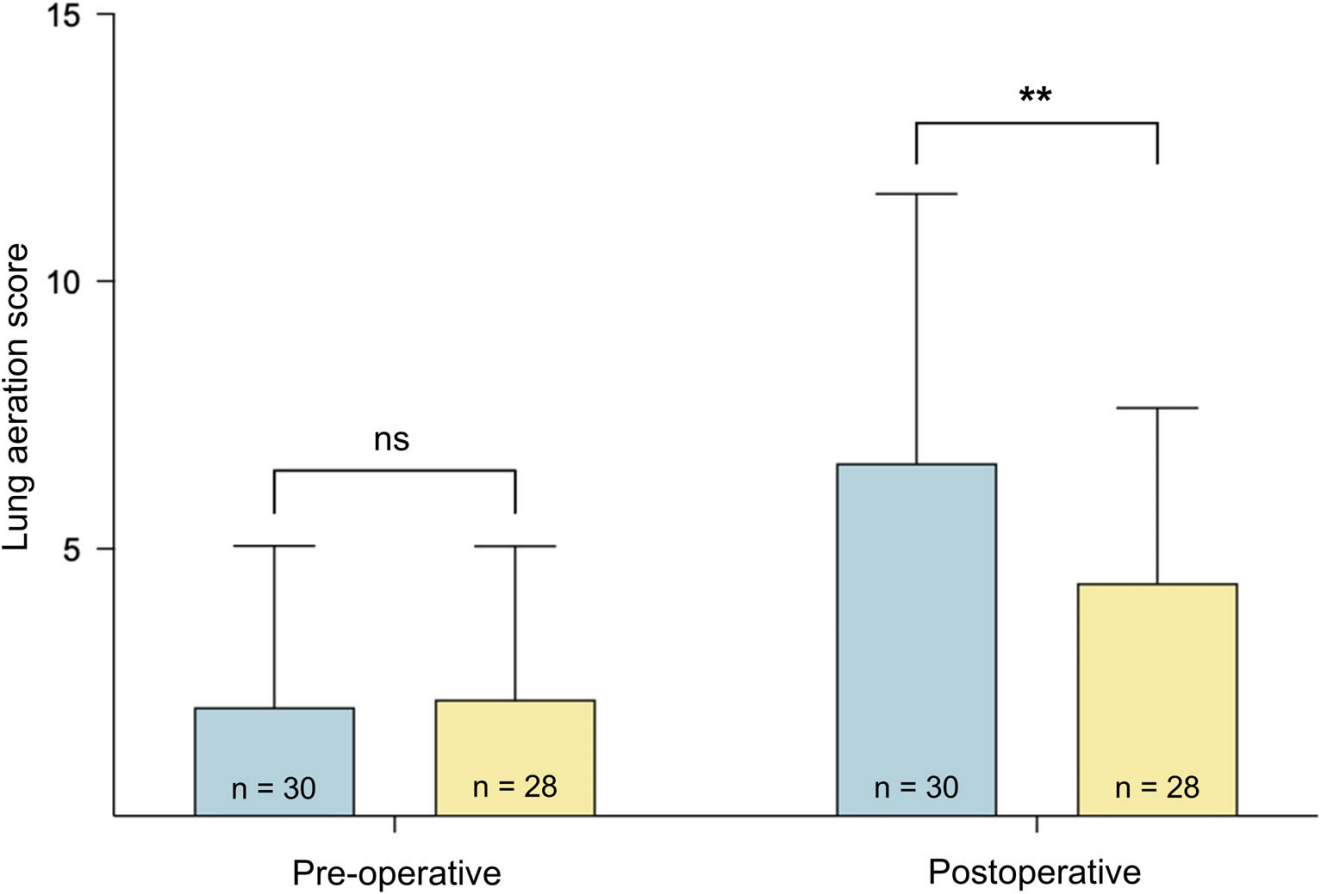
Changes in lung aeration score. Blue, group PEEP‐5; yellow, group PEEP‐BMI/3. Columns indicate mean and whiskers indicate standard deviation. **p ≤ 0.01.

A two‐way repeated measures ANOVA was employed to compare lung aeration scores, revealing a significant interaction between PEEP adjustment and time of ultrasound (pre‐ and postoperative) (F_(1, 56)_ = 9.45, p = 0.003). This indicates a greater difference between pre‐ and postoperative lung aeration scores in Group PEEP‐5 in comparison with group PEEP‐BMI/3 (Fig. 3). Consequently, a greater number of patients allocated to group PEEP‐5 (n = 7) developed atelectasis (as indicated by a newly developed lung aeration score of 3 points in any area) in comparison with group PEEP‐BMI/3 (n = 0, p = 0.011, Table 2). The absolute risk difference was 0.23 (95%CI 0.043–0.43), indicating a distinct reduction in the risk of developing atelectasis. The relative risk was ∞ (95%CI 1.98 to ∞), reflecting the complete absence of new atelectasis in the intervention group.

For postoperative measures, mean (SD) SpO_2_ on arrival in the PACU was significantly lower with PEEP‐5 compared with PEEP‐BMI/3 (92 (4) vs. 94 (3)%, respectively; p = 0.049). Thirteen patients in group PEEP‐5 and six patients in group PEEP‐BMI/3 required additional oxygen supplementation to keep postoperative SpO_2_ ≥ 90% (p = 0.036). The absolute risk reduction was 0.33 (95%CI 0.04–0.62), indicating a significant reduction in the need for oxygen supplementation in the intervention group. The relative risk for additional oxygen requirement with PEEP‐5 was 2.27 (95%CI 1.1–5). All further secondary endpoints did not show any statistically significant differences (Table 2).

## Discussion

The principal objective of this study was to evaluate the efficacy of adjusting PEEP based on BMI compared with a clinical standard PEEP of 5 cmH_2_O in improving intra‐operative respiratory mechanics and reducing postoperative loss of lung aeration.

Compared with PEEP‐5, we have shown a statistically significant reduction in driving pressure with PEEP‐BMI/3, accompanied by improved postoperative lung aeration. This was confirmed by a significantly lower increase in lung aeration score and a reduced incidence of newly developed atelectasis in patients allocated to group PEEP‐BMI/3. These improvements may have contributed to higher SpO_2_ levels on arrival in the recovery room and decreased the need for supplementary oxygen.

The selection of driving pressure as the primary outcome parameter was based on the findings of a previous meta‐analysis by Neto et al., which identified this as the sole significant mediator of the effects of protective ventilation on the development of postoperative pulmonary complications [12]. In general, reductions in driving pressure can be achieved by lower tidal volume and optimised PEEP. As the aim of this study was to analyse the effects of PEEP exclusively, both groups were ventilated with a fixed tidal volume of 7 ml.kg^‐1^ predicted body weight. The reduction in driving pressure that was observed with PEEP adjusted according to BMI was accompanied by an increased C_rs_, indicating improved respiratory system mechanics with the individualised PEEP approach. Furthermore, the absence of a significant correlation between driving pressure and BMI in patients allocated to group PEEP‐BMI/3 (in contrast with those allocated to the control group) highlights the efficacy of the BMI‐adjusted PEEP in mitigating the adverse effects of increased BMI on respiratory mechanics. Nonetheless, the relatively small between‐group difference in driving pressure, although statistically significant, suggests a modest clinical impact.

The findings of this study align with previous research advocating for individualised PEEP settings to optimise respiratory mechanics and prevent postoperative pulmonary complications [14, 21]. Conversely, studies employing a uniform PEEP setting, regardless of patient specifics, have reported uncertain benefits of higher PEEP levels, particularly in the absence of individualised adjustments [10, 11].

As the MP_el‐stat_ component of mechanical power is determined directly by the set PEEP, it was expectedly higher in group PEEP‐BMI/3. Costa et al. showed that the MP_el‐stat_ component is probably of minor importance in the development of ventilator‐associated lung injuries [33]. In terms of the elastic dynamic component of power, no significant between‐group differences were observed. Given that MP_el‐dyn_ is determined by the product of tidal volume, respiratory rate and driving pressure, we anticipated a reduction in the intervention group due to the lower driving pressure associated with BMI‐adjusted PEEP. However, given the absence of any statistically significant differences in tidal volume and respiratory rate, it can be assumed that the study lacked the necessary statistical power to detect a reduction in MP_el‐dyn_ in the PEEP‐BMI/3 group. While the results regarding respiratory mechanics were promising, neither hypotensive events nor the higher time‐weighted average of hypotensive events reached statistical significance, which may indicate the need for a larger sample size.

The pragmatic approach and easy applicability of the BMI/3 PEEP adjustment render it an appealing strategy for routine clinical practice, especially for initial PEEP adjustment. The observed improvements in lung mechanics and reduction in driving pressure could potentially translate into lower rates of postoperative pulmonary complications and better outcomes, particularly in patients with a higher BMI. However, while BMI/3 could serve as an effective ‘rule of thumb’, the resulting PEEP does not represent the optimal choice for each individual patient. The clinical relevance of the BMI‐based approach lies in its ability to provide a reasonable yet pragmatic individualisation of PEEP without requiring additional monitoring and time‐consuming PEEP titration manoeuvres. Furthermore, it could also serve as a starting point for further individualisation, if required. For example, in high‐risk patients, an initial PEEP adjustment according to BMI could be complemented by further incremental or decremental PEEP steps with careful assessment of changes in driving pressure or C_rs_ to identify the optimal PEEP for individual high‐risk patients.

Some strengths of this trial include its randomised design, automated electronic recording of ventilation and haemodynamic data and the pseudonymisation of lung ultrasound data before analysis to reduce any subjective bias. There are also a number of limitations. Although previous analyses suggest a potential causal relationship between driving pressure and postoperative pulmonary complications, this primary endpoint remains a surrogate parameter [12]. Furthermore, by not including patients with a BMI > 60 kg.m^‐2^ and the narrow BMI range of the study cohort (maximum BMI 37 kg.m^‐2^ in Group PEEP‐5 and 40 kg.m^‐2^ in Group PEEP‐BMI/3) limits the generalisability of these findings, particularly in patients living with severely obese. However, we note that previous studies suggest that the general relationship between PEEP and BMI may be consistent even in patients with higher BMI. For instance, Nestler et al. reported mean (SD) electrical impedance tomography‐derived individualised PEEP values of 18.5 (4.6) cmH_2_O in patients living with obesity, with an average BMI of 48.3 kg.m^‐2^ corresponding to a BMI/PEEP ratio of 2.6 [20]. In the study by Nestler et al., the highest electrical impedance tomography‐derived PEEP value was 25 in a patient with BMI 65 kg.m^‐2^. In a similar population of patients living with obesity, Eichler et al. found that without capnoperitoneum, the required PEEP level to maintain a positive transpulmonary pressure at end‐expiration was 16.7 cmH_2_O (95%CI 15.6–18.1), which was approximately a third of the average BMI of 48 kg.m^‐2^ [17]. Therefore, we believe that the BMI/3 formula may be applicable up to a BMI of 60 kg.m^‐2^/PEEP 20 cmH_2_O. Further studies with stratification according to BMI are required to confirm this. A further limitation is that the lack of stratification by BMI in this study resulted in a statistically non‐significant difference in BMI between the groups, with a mean (SD) BMI of 28.6 (4.4) kg.m^‐2^ in the PEEP‐5 group compared with 27.0 (5.2) kg.m^‐2^ in the PEEP‐BMI/3 group (Table 1, online Supporting Information Figures S1 and S2). Future studies might address this by including a wider BMI range, stratifying patients according to BMI and employing multicentre designs to elucidate the relationship between PEEP adjustment based on BMI and postoperative pulmonary complications across different clinical scenarios.

Although postoperative SpO_2_ on arrival in the recovery room was statistically significantly higher in the intervention group, the absolute between‐group difference in SpO_2_ was small and of a magnitude that is not clinically relevant for most patients. In addition, postoperative oxygen saturation at this stage is influenced by numerous other factors (e.g. residual anaesthetic effects, neuromuscular blockade reversal, use of opioids, smoking status and patient positioning), making it difficult to attribute differences solely to the intraoperative ventilation strategy.

In conclusion, the results of this randomised controlled trial show that adjusting PEEP based on BMI during general anaesthesia reduces driving pressure, decreases postoperative loss of lung aeration and improves both intra‐operative respiratory system compliance and postoperative oxygenation, without statistically significant negative haemodynamic effects. The BMI/3 rule offers a practical and easily applicable approach to initial PEEP adjustment, balancing respiratory benefits with haemodynamic safety. While this method provides a valuable compromise for many patients, further individualisation may be needed to achieve optimal outcomes, especially in patients at high risk of postoperative pulmonary complications. Broader implementation appears feasible, but additional studies with larger and more diverse patient populations are required to confirm its effectiveness and support definitive recommendations.

## Supporting information


**Table S1.** Modified lung aeration score.

**Figure S1.** Assignment of PEEP to BMI.
**Figure S2.** Patients categorised into cohorts of BMI > 30, 30–35 and > 35 kg.m^‐2^ and their respective group assignment.

Plain Language Summary
