# Supplementary material for: Adjustment of positive end‐expiratory pressure based on body mass index during general anaesthesia: a randomised controlled trial*
Source: Anaesthesia. 2025 Jun 23;80(11):1322–32. doi: 10.1111/anae.16656 (PMC12519930; doi:10.1111/anae.16656)
Supplement: Supplementary file 1 — Table S1. Modified lung aeration score. [file ANAE-80-1322-s001.docx]

**Table S1:** Modified Lung Aeration Score according to Monastesse et al. [1]. Each of 12 individually examined quadrants get a number of points ranging from 0-3 which are then added together with a minimal number of points 0 (no aeration loss) to 36 (complete loss of aeration).

|  | Normal Aeration | SMall Loss of aeration | moderate loss of aeration | severe loss of aeration |
| --- | --- | --- | --- | --- |
| Modified lung ultrasound score | 0 – 2 B-Lines | ≥3 B-Lines  OR  ≥ 1 small separate subpleural consolidations with normal pleural line | Multiple coalescent B-Lines  OR  small separate subpleural consolidations with irregular pleural line | Consolidations  OR  Small subpleural consolidation of >1 x 2 cm |
| Points | **0** | **1** | **2** | **3** |

**Reference**

1. Monastesse A, Girard F, Massicotte N, Chartrand-Lefebvre C, Girard M. Lung ultrasonography for the assessment of perioperative atelectasis: a pilot feasibility study. *Anesthesia and Analgesia* 2017; **124**: 494–504. https://doi.org/10.1213/ANE.0000000000001603.
