# Supplementary material for: Adjustment of positive end‐expiratory pressure based on body mass index during general anaesthesia: a randomised controlled trial*
Source: Anaesthesia. 2025 Jun 23;80(11):1322–32. doi: 10.1111/anae.16656 (PMC12519930; doi:10.1111/anae.16656)
Supplement: Supplementary file 2 — Figure S1. Assignment of PEEP to BMI. Figure S2. Patients categorised into cohorts of BMI > 30, 30–35 and > 35 kg.m‐2 and their respective group assignment. [file ANAE-80-1322-s003.pdf]

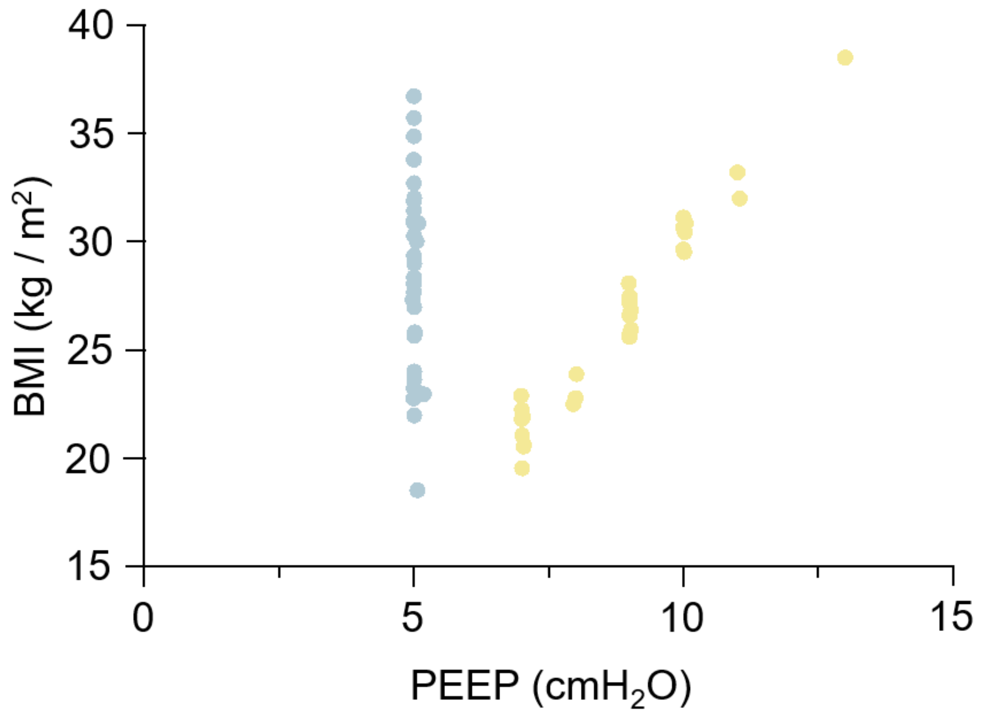

Figure S1: Illustration of the assignment of PEEP to BMI. Blue: control group; yellow: intervention group.

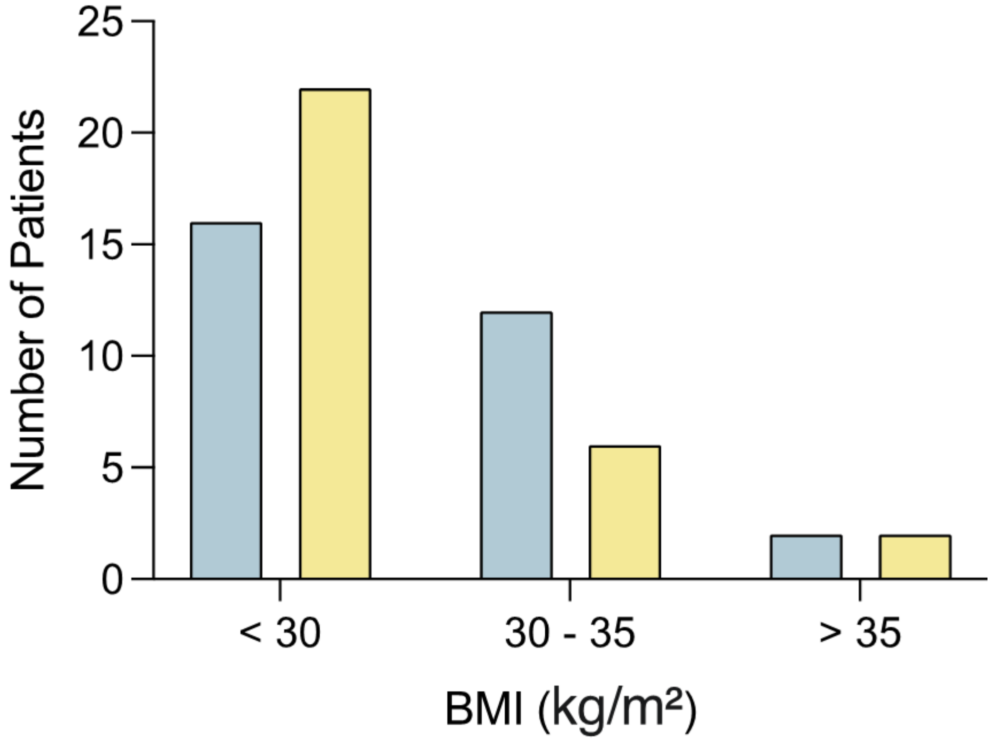

Figure S2: Number of patients categorised into cohorts of BMI >30, 30-35 and >35 and their respective group assignment. Blue: control group; yellow: intervention group.
<30: control group = 16, intervention group = 22; 30-35: control group = 12, intervention group = 6 patients; >35: two patients in each group.
