# Supplementary material for: Adjustment of positive end‐expiratory pressure based on body mass index during general anaesthesia: a randomised controlled trial*
Source: Anaesthesia. 2025 Jun 23;80(11):1322–32. doi: 10.1111/anae.16656 (PMC12519930; doi:10.1111/anae.16656)
Supplement: Supplementary file 3 — Plain Language Summary [file ANAE-80-1322-s002.docx]

**Plain Language Summary**

Protecting the lungs during surgery is important to stop problems with breathing afterwards. One way to do this is by using the right air pressure when helping someone breathe with a machine. But doctors don’t all agree on the best way to do this. This study looked at whether using air pressure based on a person’s body size (BMI) is better than using the same air pressure for everyone. We did a study with two groups of adult patients who were having surgery and needed help breathing with a tube and machine. Some patients got the same air pressure (5 cmH₂O), while others got air pressure based on their BMI (BMI divided by 3, about 7-10 cmH₂O). We used a special setting on the breathing machine that gave each person the same amount of air based on their body size. We used ultrasound to check how well their lungs were working before and after surgery. We studied 60 people. The group that got air pressure based on BMI had better results. Their lungs needed less pressure to work properly, and they had better lung movement. Their lungs also stayed more open after surgery. They needed less extra oxygen, had fewer new breathing problems, and had better oxygen levels after surgery. In people without serious lung problems, using air pressure based on BMI during surgery helped their lungs work better and kept them healthier afterward. This method is simple and can be a good way for doctors to choose the best air pressure for each patient during surgery.
